# Understanding the glioblastoma immune microenvironment as basis for the development of new immunotherapeutic strategies

**DOI:** 10.7554/eLife.52176

**Published:** 2020-02-04

**Authors:** Ana Rita Pombo Antunes, Isabelle Scheyltjens, Johnny Duerinck, Bart Neyns, Kiavash Movahedi, Jo A Van Ginderachter

**Affiliations:** 1 Myeloid Cell Immunology LabVIB Center for Inflammation ResearchBrusselsBelgium; 2 Lab of Cellular and Molecular ImmunologyVrije Universiteit BrusselBrusselsBelgium; 3 Department of NeurosurgeryUZ BrusselsBrusselsBelgium; 4 Department of Medical OncologyUZ BrusselsBrusselsBelgium; The Francis Crick InstituteUnited Kingdom; PfizerUnited States

**Keywords:** glioblastoma, microenvironment, immunotherapy, tumor-associated macrophage, tumor-associated dendritic cell, regulatory T cell

## Abstract

Cancer immunotherapy by immune checkpoint blockade has proven its great potential by saving the lives of a proportion of late stage patients with immunogenic tumor types. However, even in these sensitive tumor types, the majority of patients do not sufficiently respond to the therapy. Furthermore, other tumor types, including glioblastoma, remain largely refractory. The glioblastoma immune microenvironment is recognized as highly immunosuppressive, posing a major hurdle for inducing immune-mediated destruction of cancer cells. Scattered information is available about the presence and activity of immunosuppressive or immunostimulatory cell types in glioblastoma tumors, including tumor-associated macrophages, tumor-infiltrating dendritic cells and regulatory T cells. These cell types are heterogeneous at the level of ontogeny, spatial distribution and functionality within the tumor immune compartment, providing insight in the complex cellular and molecular interplay that determines the immune refractory state in glioblastoma. This knowledge may also yield next generation molecular targets for therapeutic intervention.

## Introduction

During the past decade, immunotherapy of cancer has reached the status of being one of the most effective cancer therapies for defined tumor types. The main progress came from immune checkpoint blockers (ICB), monoclonal antibodies that inhibit the function of molecules involved in downregulating T-cell activation such as CTLA-4 or PD-1. ICB has shown the spectacular potential of curing late stage metastatic patients with highly immunogenic tumors such as melanoma, Merkel cell carcinoma or microsatellite instability (MSI)-high cancers, largely explaining its success. However, the majority of patients, even in responsive tumor types such as melanoma, do not benefit from ICB. Even more troublesome, some tumor types have shown nearly complete refractoriness to ICB, for as yet not fully defined reasons.

Glioblastoma (GBM), the highest-grade, most prevalent and most aggressive glial tumor, is one of the cancers in which ICB has met little success so far. Several underlying mechanisms could be responsible for this failure, including the inherently heterogenous nature of this tumor type within individuals and the establishment of an immunosuppressive tumor microenvironment.

**Figure 1.**
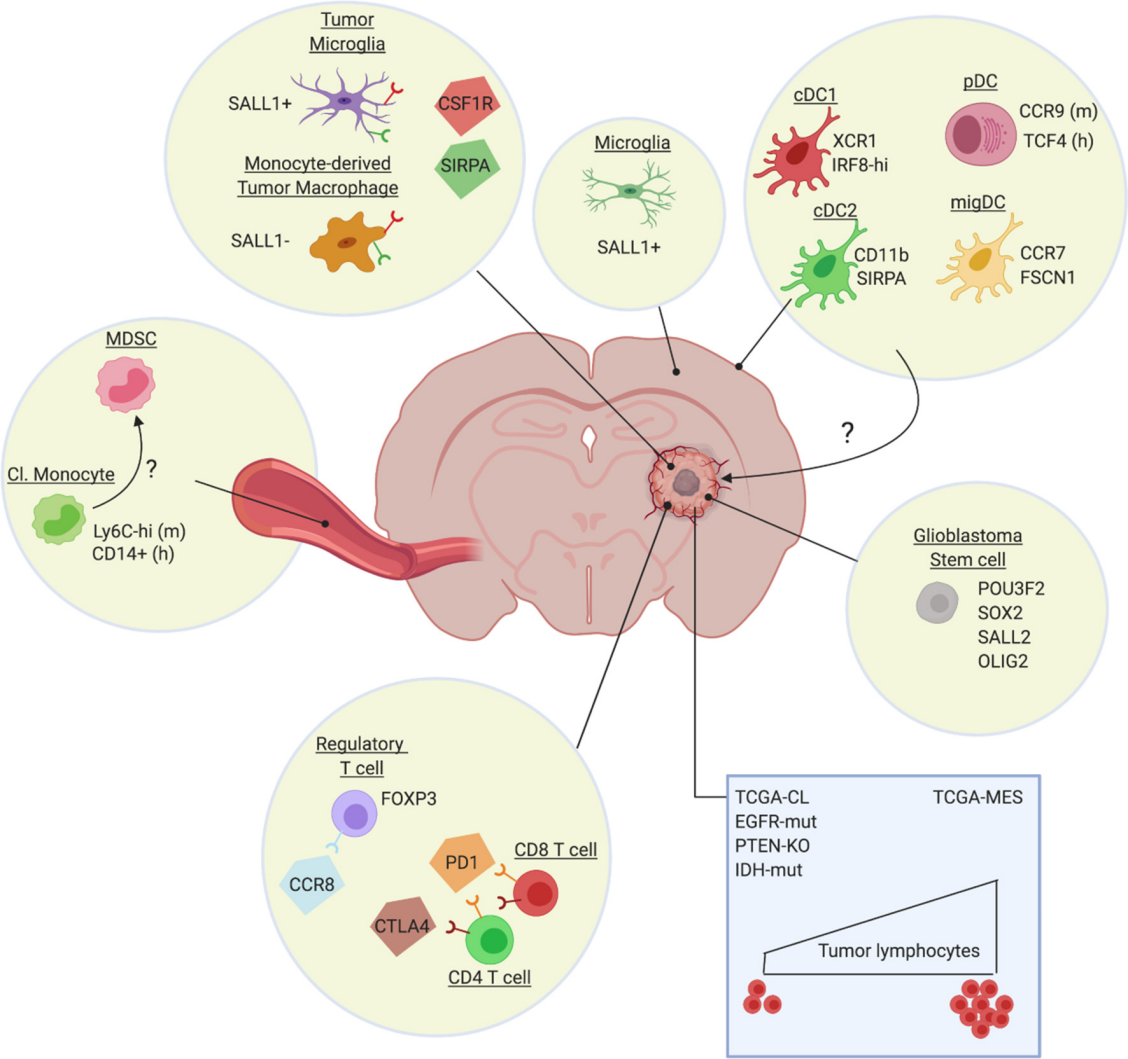
Heterogeneity of the glioblastoma immune microenvironment and potential therapeutic targets. Within glioblastoma tumors reside ontogenically distinct, immunoregulatory macrophages (Sall1^+^ tumor microglia, Sall1^-^ monocyte-derived macrophages), immunosuppressive Treg (eg CCR8^+^) and dysfunctional T-cell populations (CTLA-4/PD-1^hi^). Not much is known about intratumoral DC subsets, although distinct DC populations are found in other brain regions, such as the dura mater (Van Hove et al., 2019). Glioblastoma also affects the phenotype of classical monocytes (Cl. Monocyte) in the periphery, which acquire an immunosuppressive (MDSC-like?) phenotype. Notably, the genetic make-up of the cancer cells (blue rectangle) and potentially also of the glioblastoma stem cells, affect the immune composition of the tumor, with for example a higher presence of lymphocytes in TCGA-MES tumors. Several potential therapeutic targets (CSF1R, SIRPa, CCR8, PD-1, CTLA-4), either already tested in the clinic or promising for the future, are highlighted.

Growth of GBM tumors, but also resistance to radiotherapy and chemotherapies, is mediated by stem-like cells, whose tumor-propagating nature is fully regulated by a core set of neurodevelopmental transcription factors such as POU3F2, SOX2, SALL2, and OLIG2 (Suvà et al., 2014) (Figure 1). Various markers have been suggested for glioblastoma stem cells (Lathia et al., 2015), but it is unclear at present whether different subpopulations of GBM stem cells exist and whether these give rise to tumors with a different cellular composition. In any case, expression profiling of GBM tumors identified at least three GBM subtypes: proneural (TCGA-PN), classical (TCGA-CL) and mesenchymal (TCGA-MES) (Verhaak et al., 2010; Wang et al., 2017), which tend to differentially associate with abnormalities in PDGFRA, IDH1, EGFR and NF1 (Verhaak et al., 2010). This level of heterogeneity is dramatically increased by the notion that different GBM subtypes can be found within the same tumor and are dynamic in function of time or in response to therapy (Sottoriva et al., 2013; Patel et al., 2014; Wang et al., 2017). More recent high-resolution single-cell RNA sequencing provided even more granularity to the concept of intra-tumoral heterogeneity by identifying four cellular states for glioblastoma cells: mesenchymal-like (MES-like), astrocyte-like (AC-like), oligodendrocytic precursor cell-like (OPC-like) and neural progenitor cell-like (NPC-like) (Neftel et al., 2019). There is a preponderance of particular states in each TCGA tumor type, with TCGA-CL and TCGA-MES being enriched in AC-like and MES-like states, respectively, and TCGA-PN encompassing both OPC-like and NPC-like states. Notably, some genetic alterations favor specific cellular states, with for example *EGFR* overexpression driving an AC-like program (Neftel et al., 2019). Finally, non-genetic heterogeneity within GBM tumors is determined by the relative proximity of cancer cells to blood vessels, with mTOR activity being upregulated in the few cell layers closest to the vessels (Kumar et al., 2019). In these cells, mTOR conveys superior invasive and migratory capabilities and resistance to therapy. Together, this highly heterogeneous nature of GBM strongly undermines the efficacy of therapy, considering the likely presence of cancer cell clones which are able to escape.

In addition, defects in anti-tumor T-cell responses are commonly observed in GBM, suggesting the active induction of immunosuppression. In this respect, intracranial tumors of various histological origins (not only GBM) cause an entrapment of T cells in the bone marrow due to a loss of surface spingosine-1-phosphate receptor 1 (S1P1) (Chongsathidkiet et al., 2018). Why only intracranial tumors induce this defect and via which mechanism is currently unknown, but a reversal of this deficiency enables an enhanced migration of T cells into GBM tumors. Along the same line, thymic involution in GBM tumor-bearers results in diminished T-cell production and, hence, reduced availability of T cells for anti-GBM immunity (Andaloussi et al., 2008). Moreover, once T cells do get inside the GBM tumor, they are likely to become dysfunctional via various mechanisms (Woroniecka et al., 2018a). Recent studies provide evidence for the enriched presence of exhausted T cells within GBM tumors, with tumor-infiltrating lymphocytes (TILs) more often co-expressing the immune checkpoint molecules PD-1, LAG3 and TIM-3 than peripheral blood cells of patients or healthy controls (Woroniecka et al., 2018b; Davidson et al., 2019). Interestingly, PD-1^+^ LAG3^+^ TIM-3^+^ T cells showed impaired T-cell functions, while single positive cells for any of these markers did not. A second prominent mechanism of T-cell dysfunctionality is tolerance, mediated by the presence of suppressive cells such as regulatory T cells (Treg) and tumor-associated macrophages (TAM). These aspects will be discussed in detail in this review.

### Immunotherapeutic approaches in glioblastoma

Previous reviews have described the attempts to incorporate ICB in the treatment of GBM (Romani et al., 2018; Simonelli et al., 2018). In summary, preclinical models have provided a proof-of-concept that ICB with anti-CTLA-4 and/or anti-PD-1, either as monotherapy or in conjunction with standard-of-care treatments, improves tumor outcome. This has set the stage for multiple clinical trials in glioblastoma patients, the results of which are up to this date mostly unavailable. Here, we will focus on the most recent progress in this field. One of the most noticeable advances comes from the realization that the timing of ICB administration could majorly impact its effect. Classically, ICB is given in an adjuvant setting, improving recurrence-free survival and overall survival after surgery in cancer types such as melanoma. Neoadjuvant (pre-operative) therapy has proven to be advantageous in breast, bladder and other types of cancer, but this finding remained restricted to chemo- and radiation therapy with very little data available on ICB neoadjuvant therapy until recently (O'Donnell et al., 2019). Liu et al. (2016) demonstrated increased anti-tumor immune responses and survival upon neoadjuvant immunotherapy, in two mouse models of triple-negative breast cancer. This finding has now been reproduced in several other preclinical models (O'Donnell et al., 2019), and importantly, also in the clinical setting of resectable melanoma (Blank et al., 2018; Amaria et al., 2018; Rozeman et al., 2019) and glioblastoma (Cloughesy et al., 2019; Schalper et al., 2019). In resectable glioblastoma, either as primary or recurrent tumor, one neoadjuvant injection of anti-PD-1 monoclonal antibodies, followed by surgical resection of the tumor and repeated adjuvant anti-PD-1 treatment, resulted in a significant increase in overall survival and progression-free survival compared to adjuvant treatment alone (Cloughesy et al., 2019; Schalper et al., 2019). Remarkably, the same conclusions were reached by two independent research groups that used a different monoclonal antibody, either pembrolizumab or nivolumab. Both studies also demonstrated an intratumoral immunomodulatory effect of the neoadjuvant treatment, with an increased interferon response, production of chemokines, and signs of increased TIL activity. However, larger-scale randomized trials should be performed to fully prove the benefit of this neoadjuvant approach.

A second level of innovation comes from the assessment of novel combination therapies in preclinical models, whereby anti-PD-1 efficacy is boosted by a concurrent administration of chemotherapy (Park et al., 2019), radiation therapy (Rajani et al., 2018), other immune checkpoint blocking monoclonal antibodies (Wu et al., 2019), cancer cell-directed immunotoxins (Chandramohan et al., 2019), and nanocarriers that improve the shuttling of ICB across the blood-brain barrier (Galstyan et al., 2019). Most of these studies show a clear benefit of combining different approaches to increase anti-GBM immunity. Indeed, monotherapies usually have little impact, highlighting the strongly immunosuppressive nature of the GBM tumor microenvironment (TME). A novel avenue to increase the immunogenicity of the GBM TME comes from virotherapy, whereby oncolytic viruses not only cause a direct killing of highly proliferative cancer cells but also trigger an (innate) immune response (Iorgulescu et al., 2018). For example, a recent clinical trial applied intratumoral injections of a polio-rhinovirus chimera and could achieve a remarkable 21% of longer term survivors (at least 2 to 3 years), while <4% of such patients is observed in control groups (Desjardins et al., 2018). In a phase I study with DNX-2401, a tumor-selective replication-competent oncolytic adenovirus, 20% of patients survived >3 years from treatment, and three patients had a ≥ 95% reduction of the tumor, which was probably due to direct oncolytic effects of the virus followed by elicitation of an immune-mediated antiglioma response (Lang et al., 2018). A passive transfer of anti-tumor T-effector cells could be an alternative strategy to attack GBM tumors, although such T cells should then be able to withstand the suppressive TME. Novel generations of CAR T-cells are being generated that should show improved functionality in the TME, including the TME from GBM (Petersen and Krenciute, 2019). Finally, the adoptive transfer of antigen-presenting dendritic cells (i.e. DC vaccination) has yielded some efficacy in improving the median overall survival in clinical trials (Srivastava et al., 2019) and further research is warranted to improve the treatment regimens and the choice of the most optimal DC type and the most optimal tumor antigens. Neoantigens are the most tumor-specific and arguably the most promising antigen candidates, that show efficacy as vaccine candidates in glioblastoma patients (Keskin et al., 2019; Hilf et al., 2019).

### Heterogeneity of the tumor immune microenvironment in glioblastoma

The immune contexture of human tumors, referring to the exact composition of distinct immune cell types within tumors, changes at each tumor stage and is known to have an impact on clinical outcome (Fridman et al., 2012; Bindea et al., 2013). Hence, understanding the tumor (immune) microenvironment has recently been recognized as one of the future challenges to improve therapy of brain tumors (Aldape et al., 2019). Glioblastoma tumors are known to contain a variety of immune cell types, but with a dominance of immunosuppressive cells. Recent findings further finetune this concept through the identification of distinct subpopulations within the immunoregulatory macrophage compartment of tumors, but also distinct Treg and dendritic cell subsets have been identified and will be discussed in this review.

### Correlation between tumor genotype and the glioblastoma immune microenvironment

As mentioned earlier, innovative techniques such as extensive exome and transcriptome sequencing have revealed the existence of molecularly distinct proneural, mesenchymal and classical subtypes of glioblastoma (Verhaak et al., 2010; Wang et al., 2017). In addition, a diverse array of recurrent genomic mutations were found in these tumors, including *TP53, IDH1*, *NF1, PTEN*, *PDGFRA*, *EGFR* and MAPK pathway mutations, with particular mutations being enriched in particular glioblastoma subtypes.

Of importance in the context of ICB immunotherapy, is the observation that the number of immune cells in the human glioblastoma microenvironment appears to be associated with the predominant genetic alteration (Figure 1). TILs are enriched in TCGA-MES glioblastomas and strongly associated with mutations in *NF1* and *RB1*. Conversely, TILs are depleted in the TCGA-CL class, *EGFR*-amplified, and homozygous *PTEN*-deleted tumors and rare in histologies characterized by these alterations (Rutledge et al., 2013). In addition, the *IDH* mutant status in glioblastoma tumors significantly associates with TIL infiltration. The *IDH*-wt status is associated with significantly higher TIL infiltration and PD-L1 expression (Berghoff et al., 2017), while *IDH* mutations result in the opposite phenotype, that is a reduced IFNγ signature and reduced presence of CD8^+^ and CD4^+^ cells (Kohanbash et al., 2017; Luoto et al., 2018). Nevertheless, patients bearing *IDH*-mutated tumors have a more favourable outcome (Ceccarelli et al., 2016), suggesting that other cancer cell-intrinsic or -extrinsic mechanisms are at play beyond the presence of TILs.

One of those mechanisms could be the presence of immunoregulatory macrophages and neutrophils. In a study comparing a small number of treatment-naive *IDH*-mutated and *IDH*-wt glioblastoma tumors, *IDH-*mutants were shown to contain less macrophages which displayed a more pro-inflammatory M1-like activation state, possibly contributing to the longer survival of these patients (Poon et al., 2019). The reduced presence of macrophages in *IDH*-mutant, but also *CDK4*-amplified tumors, was also demonstrated via a computational analysis of a large number of TCGA RNAseq samples (Luoto et al., 2018). Hence, although *IDH*-mutated tumors contain a lower number of T lymphocytes, these cells may be more optimally activated due to the relative paucity of immunosuppressive macrophages and neutrophils. The frequency of macrophages and neutrophils is generally increased in TCGA-MES tumors versus the other TCGA types (Wang et al., 2017). Remarkably, genes for both tumor-promoting M2-like as well as potentially anti-tumoral M1-like macrophages were enriched in TCGA-MES tumors, in particular in the *NF1*-deficient cases (Wang et al., 2017; Luoto et al., 2018). The absence of *NF1* appears to result in chemoattraction of macrophages via an as yet undefined mechanism. In addition, high B-cell and CD4^+^ T-cell components were commonly observed in TCGA-MES samples, while immune cell components tend to be low in TCGA-PN samples (Luoto et al., 2018). In general terms, computational analysis grouped glioblastoma tumors into three immune response-related subgroups, termed negative (defined by a relative paucity of immune cells; enriched by TCGA-PN and *CDK4-MARCH9* amplification), humoral (defined by a high B-cell and CD4^+^ T-cell compartment; enriched by TCGA-MES) and cellular-like (defined by a higher ‘negative regulation of T-cell activation’ and ‘gamma delta T-cell’ cluster; enriched by TCGA-CL and samples with a high macrophage content) (Luoto et al., 2018).

The question remains how these findings relate to the responsiveness of patients to ICB. Surprisingly, in spite of the immunologically different tumor microenvironment in TCGA subtypes, no association was found with ICB responsiveness (Zhao et al., 2019). Rather, non-responding patients bear significantly more *PTEN* mutations, which are associated with immunosuppressive gene expression signatures, while responders are enriched in MAPK pathway alterations (*PTPN11, BRAF*). These findings illustrate that responsiveness to ICB is of a greater complexity than the mere presence of immune cell types within the microenvironment.

### Impact of standard-of-care treatments on the Glioblastoma immune microenvironment

Long-standing treatment options for glioblastoma include surgery, radiotherapy and temozolomide chemotherapy, all of which may affect the immune composition of glioblastoma tumors. In addition, brain oedema is most often treated with dexamethasone, a steroid with known immunoregulatory capacities.

Radiation therapy obviously aims to eliminate the proliferating cancer cells, but one of its side effects is the induction of hypoxia. In turn, hypoxia initiates a lethal cascade of events consisting of the increased production of CXCL12, the subsequent recruitment of CXCR4 and CXCR7-positive bone marrow-derived monocytes and hematopoietic progenitor cells that differentiate into tumor-promoting macrophages and mediate vasculogenesis and tumor recurrence in mice (Tabatabai et al., 2006; Kioi et al., 2010; Tseng et al., 2011). Hence, blockade of CXCL12 (Liu et al., 2014) and CXCR7 (Walters et al., 2014) delays recurrence of tumors upon irradiation in preclinical models. Most importantly, a recent Phase I/II clinical trial with the reversible CXCR4 inhibitor plerixafor – a strategy named Macrophage Exclusion after Radiation Therapy or MERT – in conjunction with chemoirradiation improved local control of tumor recurrence in newly diagnosed glioblastoma patients (Thomas et al., 2019). Another effect of radiation is the upregulation of PD-L1 at the surface of tumor-infiltrating myeloid cells. This effect has been exploited to target anti-PD-L1-functionalized lipid nanoparticles, carrying toxic compounds, to these myeloid cells in conjunction with irradiation, leading to an improved anti-tumor response in mice (Zhang et al., 2019).

Temozolomide is the most widely used chemotherapy for patients with glioblastoma (GBM), whose action not only directly affects cancer cells but also depends on its immunomodulatory properties (Karachi et al., 2018). Temozolomide induces lymphopenia, which, counterintuitively, can be harnessed to improve immunotherapy. Indeed, lymphoablative doses of temozolomide were shown to increase tumor antigen-specific immune responses in GBM patients (Sampson et al., 2011; Batich et al., 2017) and GBM-bearing mice (Sanchez-Perez et al., 2013). Mechanistically, it was suggested that compensatory homeostatic cytokines after temozolomide therapy cause enhanced immune responses by reduction of the T-cell activation threshold and proliferation induction.

In recent years, it became clear that dexamethasone treatment of patients may strongly affect outcome, with a lower overall survival in recurrent glioblastoma patients (Wong et al., 2015). This adverse effect is most likely due to the well-known immunomodulatory function of steroids. Indeed, glioblastoma patients treated with dexamethasone contained significantly less lymphocytes in their blood, but more myeloid cells with potentially suppressive functions (Chitadze et al., 2017).

### Heterogeneity of macrophages within the Glioblastoma immune microenvironment

Macrophages are a large component of many tumor types and have been an attractive target for glioblastoma therapy (Pyonteck et al., 2013; Quail et al., 2016). Originally, TAMs were often thought to exclusively originate from tumor-infiltrating monocytes, but both in CNS and peripheral tumor types a fraction can be derived from the tissue-resident macrophage pool (Movahedi and Van Ginderachter, 2016; Laviron and Boissonnas, 2019). Recent work that has relied on single-cell RNA sequencing combined with fate mapping approaches has highlighted the rich diversity of murine tissue-resident brain macrophages. (Van Hove et al., 2019) (Figure 1). In this respect, parenchymal microglia differ from macrophages located in the brain's border regions, such as the meninges and the choroid plexus. Whether border-associated macrophages contribute to glioblastoma or metastatic brain tumors is not known. However, there is now firm evidence that TAMs in GBM tumors are partly derived from embryonic microglia (Figure 1). Tumor-associated microglia and monocyte-derived macrophages can be distinguished via genetic lineage tracing, which was used to sort these populations from transplantable and spontaneous mouse models of glioblastoma followed by RNA-sequencing (Bowman et al., 2016; Chen et al., 2017). These data revealed that the glioblastoma microenvironment instructs the transcriptional landscape of these cells, with prominent differences between microglia and monocyte-derived TAMs at multiple levels, including differential expression of genes involved in IL-1 signaling, chemokine and inflammatory cytokine production and antigen presentation. Whether this transcriptional divergence is reflected at the functional level and whether these subsets differentially affect glioblastoma progression is not known, but seems likely. Indeed, tissue-resident interstitial macrophages and their monocyte-derived counterparts are also found in mouse lung tumors, where resident TAMs are suggested to support cancer cell expansion, while monocyte-derived cells might contribute to cancer cell dissemination (Loyher et al., 2018). Also in mouse mammary carcinoma, a distinction was made between monocyte-derived TAMs and resident mammary tissue macrophages, with only the former contributing to a suppression of anti-tumor cytotoxic T-cell responses (Franklin et al., 2014).

In glioblastomas, monocyte-derived macrophages tend to be more enriched in the tumor core, while microglia-derived TAM are typically found at the tumor periphery (Chen et al., 2017). Live in vivo 2-photon microscopy confirmed the distinction between the two TAM populations in the mouse and demonstrated that monocyte-derived cells are small and motile, while microglia are large sessile cells whose processes are continuously extending and retracting within tumors (Chen et al., 2019a). Importantly, the existence of microglia and monocyte-derived TAM populations has now also been shown in human GBM (Müller et al., 2017; Darmanis et al., 2017). However, it will be interesting to evaluate whether microglia and monocyte-derived TAM further consist of specialized subpopulations, for example instructed by their precise intratumoral localization, as was demonstrated for TAM in other tumor models (Movahedi et al., 2010; Casazza et al., 2013). For this reason, future single-cell RNA-sequencing efforts will provide crucial new insights, and will undoubtedly allow the identification of multiple TAM subsets, as was already shown in mouse and human lung tumors (Zilionis et al., 2019). An interesting possibility is that microglia- and monocyte-derived TAMs may exhibit a differential ability to infiltrate or colonize specific tumor microenvironments or tumor macrophage niches.

Interesting novel findings in preclinical models provide further insights in the molecular mechanisms that govern the attraction of monocyte-derived macrophages to the glioblastoma microenvironment. Cancer cell- and host cell-derived osteopontin mediates macrophage infiltration through the interaction with integrin α_v_β_5_ and instructs a pro-tumoral phenotype in these cells (Wei et al., 2019). Also the Aryl Hydrocarbon Receptor (AHR) is indirectly implicated in recruiting monocytes to the tumor environment by driving CCR2 expression, the receptor for the major monocyte chemoattractants CCL2 and CCL7 (Takenaka et al., 2019). In *PTEN-*deficient tumor models, enhanced LOX production is responsible for the enhanced attraction of macrophages via the β1 integrin-PYK2 pathway, which subsequently promote tumor growth via SPP1 (Chen et al., 2019b). However, how this finding correlates with the enhanced responsiveness of *PTEN-*mutant tumors to ICB therapy in patients is not clear and requires further investigation, including the potential differences between the *PTEN* mutations found in patients and the *PTEN*-null phenotype in mouse models.

Another point of interest is the systemic influence of brain tumors on peripheral myeloid cells. GBM-secreted IL-6 induces immunosuppressive myeloid cells in the periphery that express high levels of PD-L1 in orthotopic mouse models (Lamano et al., 2019). This finding is in line with CyTOF fingerprinting of patients’ peripheral blood, indicating a significant elevation of myeloid-derived suppressor cells (MDSC), but not Treg in the circulation (Alban et al., 2018) (Figure 1). Hence, the myeloid cells that infiltrate GBM tumors may have already been primed into a tumor-promoting role. The importance of MDSC, both the monocytic and granulocytic subtype (Movahedi et al., 2008), is further confirmed by the finding that their presence in mouse GBM tumors is associated with a reduction in the number of tumor-infiltrating lymphocytes (Raychaudhuri et al., 2015). Moreover, within the murine glioblastoma environment, MDSC were shown to induce regulatory B cells (Lee-Chang et al., 2019). Interestingly, CCL2 produced by TAM mediates the recruitment of suppressive CCR2^+^ monocytic MDSC and CCR4^+^ Treg, explaining the clinical observation that elevated intratumoral CCL2 expression levels correlate with reduced overall survival (Chang et al., 2016). Of note, recruited monocytic MDSC may in turn differentiate into TAM. In a preclinical setting, this notion has now been taken further by showing that a small molecule CCR2 antagonist sensitizes otherwise resistant murine gliomas to ICB therapy (Flores-Toro et al., 2020).

### Heterogeneity of regulatory T cells (Treg) within the glioblastoma immune microenvironment

The presence of Treg has been amply described in multiple cancer types, but their value as predictors of disease outcome is debatable in glioblastoma. Independent researchers described an increased presence of FOXP3^+^ Treg in higher tumor grades of various brain tumor types, including glioblastoma (El Andaloussi and Lesniak, 2007; Jacobs et al., 2010). However, the correlation of Treg with decreased survival, which was anticipated based on the T-cell suppressive capacity of these cells, has been very moderate at best (Jacobs et al., 2010; Yue et al., 2014; Thomas et al., 2015). Nevertheless, Treg-directed therapies, such as agonist anti-GITR or anti-LAP (Latency-associated Peptide) mAb treatment, have shown some promise in mouse glioblastoma models (Miska et al., 2016; Patel et al., 2016; Gabriely et al., 2017).

A potential reason for the unclear association of Treg with disease outcome could be the existence of distinct Treg subsets which may differ in their suppressive capacity and whose presence in tumors may vary between individual patients. Early preclinical work suggested that the majority of Tregs infiltrating glioblastoma tumors are thymus-derived (Wainwright et al., 2011), although it seems likely that the local de novo induction of Treg in the tumor microenvironment is a possibility as well. As a matter of fact, indirect evidence based on correlative studies proposed glioblastoma-induced PD-L1 expression as a mechanism of Treg induction and maintenance in patients (DiDomenico et al., 2018). The attraction of these Treg to the tumor microenvironment may be mediated by various mechanisms, including IDO activity (Wainwright et al., 2012) and CCL2 production, which interacts with CCR4 at the surface of Treg (Chang et al., 2016). Interestingly, another chemokine receptor, *CCR8*, has recently been identified as a marker that is specifically expressed in at least a subset of tumor-infiltrating Treg, but not Treg in the periphery, as part of a tumor-infiltrating Treg transcriptional signature that is conserved across species and tumor types (Plitas et al., 2016; De Simone et al., 2016; Zheng et al., 2017; Magnuson et al., 2018) (Figure 1). CCR8^+^ Treg were reported as very potent suppressors, suggesting that this Treg subset is likely important in the creation of an immunosuppressive tumor microenvironment (Barsheshet et al., 2017). However, to what extent the tumor-infiltrating Treg signature and CCR8^+^ Treg are present within glioblastoma tumors is currently unknown.

Another marker that is functionally important on Treg within tumors is Neuropilin-1 (Nrp1). The interaction of Nrp1 with its ligand Semaphorin 4a was shown to stabilize the Treg phenotype in the mouse, which turns out to be especially important for the suppression of anti-tumor immune responses (Delgoffe et al., 2013). The absence of Nrp1 in Treg leads to their loss of suppressive potential but also to their production of IFNγ, which subsequently annihilates the suppressive capacity of neighbouring Nrp1^+^ Treg in murine tumors (Overacre-Delgoffe et al., 2017). This mechanism may be stimulated by enhanced HIF-1α expression in Nrp1-deficient Treg. Interestingly, HIF-1α expression in hypoxic tumor-infiltrating Treg was indeed shown to decrease the intrinsic suppressive activity of these Treg, but was at the same time required for the migration of Treg into mouse glioblastoma tumors (Miska et al., 2019). Consequently, the absence of HIF1α in Treg leads to the reduced growth of glioblastoma tumors.

Altogether, suppressive Treg subsets are induced in the microenvironment of multiple tumor types. As these cells could be prime targets for therapeutic intervention, it will be important to assess their presence and relevance in glioblastoma.

### Heterogeneity of dendritic cells within the Glioblastoma immune microenvironment

Recent findings, in other tumor types than glioblastoma, have uncovered the existence of distinct DC populations in solid tumors. Indeed, at least three types of conventional DCs (cDCs), in addition to plasmacytoid DCs (pDCs), are present in the tumor microenvironment of multiple mouse and human tumors (Laoui et al., 2016; Roberts et al., 2016; Zilionis et al., 2019). The functionality of these cells is being explored in mouse tumor models. cDC1 are rare cells within tumors that were repeatedly reported to migrate to the tumor-draining lymph nodes where they cross-present tumor antigens to CD8^+^ T cells (Broz et al., 2014; Roberts et al., 2016; Laoui et al., 2016). Interestingly, NK cells attract cDC1 to the tumor environment via the secretion of various growth factors and chemokines, such as Flt3L, CCL5 and XCL1 (Böttcher et al., 2018; Barry et al., 2018). cDC2, which are typically more abundant within tumors than cDC1, also have the capacity to drive T-cell responses, but are more directed towards the activation of CD4^+^ T cells. As such, these cells were shown to initiate a Th17 response (Laoui et al., 2016), but their full T-cell activating potential may only be unleashed in the absence of Treg (Binnewies et al., 2019). Both mouse and human lung tumors also harbor an additional DC subset that exhibits a migratory gene signature (Zilionis et al., 2019), although this cannot be taken as proof for actual migration.

Translating these findings to glioblastoma is not trivial, as a systematic analysis of DC subpopulations in these tumors is currently lacking. Recent work has shown that various dendritic cell subsets are present in the murine homeostatic brain (Mrdjen et al., 2018; Van Hove et al., 2019), where they are thought be critical for initiating neuroinflammation (Mundt et al., 2019; Jordão et al., 2019). In the context of glioblastoma, one study reported that CCR2^+^ Lin^-^ hematopoietic stem and progenitor cells (HSC) can differentiate into cross-presenting DC within mouse glioblastoma tumors (Flores et al., 2018). These HSC-derived DC can be isolated from glioblastoma tumors to successfully vaccinate mice and induce anti-tumor immunity in the vaccine recipients. In addition, their presence potentiates the effect of immune checkpoint blockade immunotherapy. Along the same line, increasing the number of intratumoral CD103^+^ cDC1 through the administration of Flt3L (Miao et al., 2018), or activating them via TLR3 agonists (Garzon-Muvdi et al., 2018), magnifies the response of mouse glioblastomas to immune checkpoint blockade, suggesting the importance of this DC type to augment immunity against glioblastoma, similar to other tumor types. However, Fibrinogen-like Protein 2 (FGL2), that is predominantly secreted by the cancer cells, may interfere with the induction of these immunostimulatory DCs (Yan et al., 2019). Indeed, FGL2 interferes with GM-CSF signalling, blunting the differentiation of CD103^+^ cDC1 and consequently lowering the CD8^+^ T-cell response. Overall, it can be concluded that interventions which increase the number of CD103^+^ cDC1 in glioblastoma tumors or augment their activation state may be beneficial. Whether cDC2 can also contribute to anti-glioblastoma immunity remains to be determined.

### Perspective on novel immunotherapies directed against cells within the tumor immune microenvironment

Finally, how can the recently acquired knowledge on the heterogeneity of the glioblastoma immune microenvironment be turned into novel therapeutic avenues? An important caveat in this respect is the capacity of drugs to cross the blood-brain barrier (BBB) and to reach sufficiently high concentrations in the brain. A number of very recent studies have highlighted the potential of nanoparticles to deliver therapeutic cargo to the mouse brain. Solid lipid nanoparticles can deliver small interfering RNAs to the mouse glioblastoma microenvironment, following a low-dose irradiation to prime brain uptake (Erel-Akbaba et al., 2019). A novel tumor penetrating peptide that targets cell surface p32, LinTT1 (AKRGARSTA), has also been reported as a GBM targeting ligand for systemically-administered nanoparticles (Säälik et al., 2019). Moreover, nanoscale immunoconjugates (NICs) on a natural biopolymer scaffold, poly(β-L-malic acid), were produced with covalently attached anti-CTLA-4 or anti-PD-1 for systemic delivery across the BBB and activation of local brain anti-tumor immune responses (Galstyan et al., 2019). Notably, nanoparticles often end up being phagocytosed by tumor-associated macrophages and are, hence, interesting tools to modulate the activity of these cells. The fact that modulating the macrophage compartment is indeed a promising approach was shown a couple of years ago by the administration of a brain penetrating CSF1R inhibitor in mice, which repolarized TAM into a more anti-tumoral phenotype resulting in a reduced GBM progression (Pyonteck et al., 2013). Likewise, a blocking anti-CD47 antibody mobilizes the phagocytic capacity of both monocyte-derived and microglial TAMs in mouse glioblastoma models (Hutter et al., 2019). As a matter of fact, this type of approaches could now be combined with strategies to increase the presence of antigen-presenting cells, by the targeted delivery of growth factors or chemokines for cDCs.

Targeting tumor-infiltrating Treg, without affecting their peripheral counterparts to avoid auto-immune complications, could be another interesting approach. The recent identification of molecules, such as CCR8, that are specifically upregulated on these cells within tumors may provide novel therapeutic anchor points in multiple tumor types. It needs to be evaluated whether these tiTreg-specific molecules are implicated in Treg functionality as this will determine the type of compound we need for therapy (agonists, antagonists,...).

Overall, increasing knowledge about subpopulations of immune cells that either promote or inhibit glioblastoma tumor progression will allow more specific therapeutic approaches against this aggressive type of brain tumor.
